# *Bacillus subtilis* Strain TCX1 Isolated from *Ambrosia artemisiifolia*: Enhancing Cucumber Growth and Biocontrol Against Cucumber *Fusarium* Wilt

**DOI:** 10.3390/plants14193068

**Published:** 2025-10-04

**Authors:** Yuzhu Dong, Mengzhuo Zhu, Yingwen Zhao, Enjing Yi, Jing Zhang, Ze Wang, Chenxi Wang, Cuimei Yu, Lianju Ma

**Affiliations:** 1 College of Life Sciences, Shenyang Normal University, Shenyang 110034, China; 18811657916@163.com (Y.D.); 15566589353@163.com (M.Z.); 15004047945@163.com (Y.Z.); 17614256922@163.com (E.Y.); 18141740592@163.com (J.Z.); aca_zq@163.com (Z.W.); 2 College of Agronomy, Shenyang Agricultural University, Shenyang 110161, China; 18846646221@163.com

**Keywords:** *Ambrosia artemisiifolia*, endophyte, *Bacillus subtilis* strain TCX1, plant growth promotion, cucumber *Fusarium* wilt, antagonistic activity

## Abstract

*Fusarium* wilt disease, caused by *Fusarium oxysporum* f. sp. *cucumerinum* (*FOC*), leads to widespread yield losses and quality deterioration in cucumber. Endophytes, as environmentally friendly control agents that enhance pathogen resistance in their host plants, may mitigate these problems. In this study, we isolated 14 endophytic bacteria from invasive *Ambrosia artemisiifolia* and screened the strain *Bacillus subtilis* TCX1, which exhibited significant antagonistic activity against *FOC* (inhibitory rate of 86.0%). TCX1 killed *Fusarium oxysporum* by being highly likely to produce lipopeptide and producing wall hydrolytic enzymes including protease, cellulase, and β-glucanase, thereby inhibiting mycelial growth and spore germination and causing peroxidation of *FOC*’s cytoplasmic membrane. In addition to its direct effects, TCX1 exerts indirect effects by inducing cucumber resistance to *FOC*. When cucumber seedlings were inoculated with TCX1, antioxidant enzymes related to disease resistance, including Superoxide dismutase (SOD), Peroxidase (POD), Polyphenol oxidase (PPO) and Phenylalanine ammonialyase (PAL) in cucumber, were significantly increased. The marker genes involved in induced systemic resistance and the salicylic acid signaling pathway, such as *npr1*, *pr1a*, *pr2*, *pr9*, *lox1*, and *ctr1*, were also dramatically upregulated, indicating these pathways played an important role in improving cucumber resistance. Notably, TCX1 can also promote cucumber growth through producing indole-3-acetic acid, solubilizing phosphate, and secreting siderophores. Given that TCX1 has dual functions as both a biological control agent and a biofertilizer, it offers an effective strategy for managing cucumber seedling blight while enhancing plant productivity.

## 1. Introduction

Cucumber (*Cucumis sativus* L.) is one of the most widely commercially cultivated vegetables globally and a staple fresh fruit in the diet, known for its refreshing taste and high vitamin E content. Thus, it holds significant economic and nutritional value. Given the escalating demand for cucumbers, breeding high-yielding, high-quality, and disease-resistant cucumber varieties has become a focal point in cucumber cultivation. Although conventional breeding methods have been instrumental in advancing cucumber production over the past decades, they are often hindered by long breeding cycles, inconsistent yields, and susceptibility to pests and diseases [1]. In recent years, molecular techniques have been integrated into cucumber breeding programs, resulting in substantial advancements in the selection and breeding of superior varieties, as well as an overall improvement in quality [2]. Despite these advancements, cucumber remains highly susceptible to horticultural diseases, leading to significant yield and economic losses from diseases such as *Fusarium* wilt, *Verticillium* wilt, downy mildew, and powdery mildew [3]. Among these diseases, cucumber *Fusarium* wilt, caused by *Fusarium oxysporum* f. sp. *cucumerinum* (*FOC*), is a highly destructive soil-borne disease worldwide, severely impacting cucumber yield and quality [4,5]. Infection of cucumber’s vascular system by the soilborne pathogen *FOC* gives rise to chlorosis, vascular discoloration, leaf wilting, stunted plant growth, and, when the condition is severe, premature plant mortality [6,7]. Although fungicides, crop rotation, and resistant cultivars can help mitigate the damage caused by *FOC*, these methods are often expensive, environmentally unfriendly, or insufficient in their effectiveness [8]. Therefore, the search for an economical and environmentally sustainable solution to manage cucumber *Fusarium* wilt has emerged as a pressing challenge in recent years.

The rapid development of modern agricultural production has led to the widespread use of pesticides, fertilizers, and insecticides. While these chemicals effectively control agricultural and forestry pests and diseases, a significant portion of them remains as residues in the soil and is absorbed by plants. This accumulation can ultimately jeopardize crop production, food safety, and human health [9]. As eco-friendly biocontrol agents, endophytes provide a sustainable substitute for conventional agricultural practices: they decrease the reliance on synthetic fertilizers and pesticides, and in turn lower the risks linked to chemical applications [10]. Endophytic bacteria are beneficial microorganisms that thrive within plants during certain or all stages of their life [11]. Their presence and secondary metabolites can enhance plant growth without causing any disease in host plants even under normal and challenging conditions, such as heavy metals, drought, salinity, pests, cold, and diseases [12].

*Bacillus subtilis*, a common bacterium in host plants, promotes growth and resistance to diseases, exerting numerous beneficial effects both directly and indirectly through multiple mechanisms. At the endophyte level, it assists host plants in acquiring increased amounts of limiting plant nutrients, including phosphorus, nitrogen, and iron [13,14,15]. Furthermore, as an endophyte, *Bacillus* enhance the nutrient uptake and biomass of host plants by producing growth-regulating phytohormones, such as abscisic acid, cytokinins, ethylene, gibberellins, and indole-3-acetic acid (IAA) [15,16]. The presence of 1-aminocyclopropane-1-carboxylate deaminase genes (ACC deaminase) in *Bacillus* species reduces ethylene levels in plants, thereby promoting growth and enhancing drought tolerance in the treated plants [17]. In addition, *Bacillus subtilis* protects plants from a variety of phytopathogens by producing secondary metabolites, antibiotics, and lytic enzymes. These compounds are effective against bacterial, fungal, and viral pathogens [18,19]. *Bacillus subtilis* strains DS07 and DS32, as well as *Bacillus velezensis* strain Q6, secrete lipopeptides as a means of resistance against Mulberry Zonate leaf spot disease [20]. In the interactions between endophytes and their hosts, *Bacillus* species play a key role in inducing systemic resistance (ISR) to safeguard their host plants against fungal, bacterial, and viral pathogens [21,22,23]. Studies have demonstrated their beneficial effects in controlling blue mold, nematodes, and various wilt-inducing pathogens through the salicylic acid (SA) and jasmonic acid/ethylene (JA/ET) signaling pathways [24,25]. Interestingly, bacterial lipopeptides, specifically those produced by *Bacillus subtilis* strain CtpxS2-1, can elicit induced systemic resistance (ISR) in Andean lupin against anthracnose [26]. Similarly, systemic acquired resistance (SAR) is also effective in *Bacillus subtilis*, sharing functional similarities with ISR but differing in its dependence on the salicylic acid signaling pathway and the accumulation of pathogenesis-related proteins (PR proteins) encoded by *pr* genes. An example of this is *Bacillus subtilis* NBRI-W9, which induces tomato defense against *Fusarium chlamydosporum* NBRI-FOL7 through SAR [27]. Given the habitat specificity and significant agricultural potential of endophytes, we propose that endophyte-based biological control could be an effective strategy for managing cucumber *Fusarium* wilt.

In this study, endophytes were isolated from *Ambrosia artemisiifolia*, commonly referred to as ragweed. Ragweed, a native North American annual weed/herb belonging to the Asteraceae family, has rapidly spread across Asia and Europe [28]. It outpaces native species in terms of growth speed, ease of dissemination, and efficiency in light energy utilization, photosynthetic response mechanisms, as well as other physiological and ecological traits. This exceptional adaptability and growth advantage may be attributed to its endophytes. Therefore, we isolated 14 antagonistic endophytic bacteria from invasive *Ambrosia artemisiifolia*. Among the isolates, we identified an antagonistic strain named TCX1, which belongs to *Bacillus subtilis* and exhibits significant growth-promoting properties and antimicrobial activity in cucumber. We verified its efficacy and furthermore revealed its mechanisms of growth promotion and disease resistance. Our results strongly indicated that this strain holds significant research potential for the development of biocontrol agents.

## 2. Results

### 2.1. Isolation and Screening of Antifungal Endophytic Bacteria from Ambrosia artemisiifolia

#### 2.1.1. Isolation and Identification of Endophytic Bacteria

A total of 14 different endophytic bacteria were isolated from various tissues of *Ambrosia artemisiifolia*, including 1 strain from leaves, 5 strains from stems, and 8 strains from roots. 16S rRNA sequencing revealed that they belonged to 8 genera: *Bacillus*, *Pseudomonas*, *Xanthomonas*, *Aeromonas*, *Lysinibacillus*, *Enterobacter*, *Curtobacterium*, and *Delftia*. Notably, most of the isolates belonged to the genus *Bacillus*. The sequence similarity between the isolates and the type strains within their respective genera, as determined by 16S rRNA sequencing, is shown in Table 1.

#### 2.1.2. Detection and Screening of Potent Antifungal Endophytes from Isolated Strains

The study conducted a preliminary screening of 14 distinct endophytic bacteria isolated from ragweed against 12 prevalent and highly destructive plant pathogenic fungi. The results indicated that five strains of endophytic bacteria, namely TCX1, TCX6, TCX7, TCX12, and TCX14, demonstrated inhibitory effects against various plant pathogens (Supplementary Table S1).

To further refine the selection of antagonistic strains, these five strains underwent additional screening via plate confrontation, with the inhibition rates presented in Table 2. Statistical analysis further revealed that, among the five strains, *Bacillus subtilis* strain TCX1 exhibited the broadest range of inhibitory activity against a variety of pathogenic fungi. TCX1 demonstrated effectiveness against a total of 12 types of plant pathogens, including *Acremonium strictum*, *Fusarium graminearum*, *Cercospora zeae-maydis*, *Phytophthora capsici Leonian*, *Sclerotinia sclerotiorum*, *Bipolaris zeicola*, *Fusarium oxysporum* f. sp. *cucumerinum*, *Trichothecium roseum*, *Pythium aphanidermatum*, *Fusarium oxysporum* f. sp. *melonis*, *Fusarium culmorum*, and *Botrytis cinerea Pers*. TCX1 not only exhibited a broad inhibitory spectrum but also demonstrated a high inhibition rate against its target pathogens. The average inhibition rate reached approximately 50%, with the highest inhibition rate of 86% observed against *FOC*.

### 2.2. Plant Growth-Promoting Functions of Antifungal Bacillus subtilis Strain TCX1

**Figure 1:**
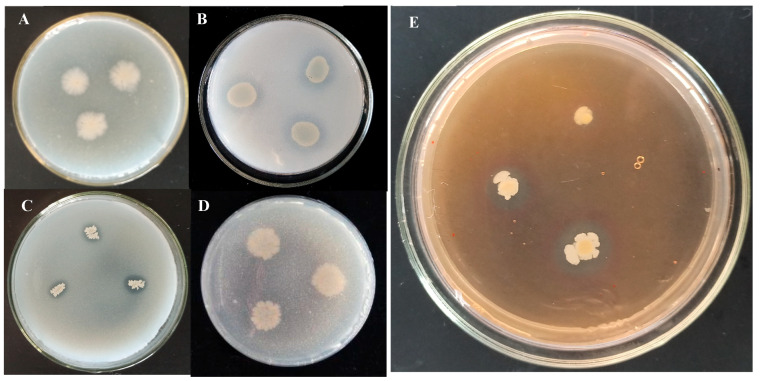
Plant-beneficial traits of *Bacillus subtilis* strain TCX1 isolated from *Ambrosia artemisiifolia*. (**A**) Organic phosphorus-solubilizing ability of strain TCX1. TCX1 was inoculated onto organophosphorus yolk medium solid plates, and the plates were incubated at 37 °C for 7 d to assess its organic phosphorus-solubilizing ability. (**B**) *Pseudomonas monteilii* YDX-22 was inoculated onto organophosphorus yolk solid medium as the positive control for organic phosphorus-solubilizing activity. (**C**) The inorganic phosphorus-solubilizing ability of TCX1 identified by PKO (Pikovskaya Medium). (**D**) *Bacillus aryabhattai* N7 was inoculated onto PKO medium as the negative control for inorganic phosphorus-solubilizing activity. (**E**) Capacity of TCX1 to produce siderophores. The experiments were repeated three times, and representative images are shown.

To investigate the growth-promoting activity of TCX1, we screened TCX1 for IAA production. When the culture supernatant of TCX1 was mixed with Salkowski’s reagent, a pink coloration was observed, indicating IAA production. Quantitative analysis revealed that TCX1 produced 1.097 ± 0.3 mg·L^−1^ of IAA. In addition, the phosphate-solubilizing ability is a crucial indicator for evaluating the growth-promoting potential of endophytes. PKO medium is designed to assess inorganic phosphorus-solubilizing ability, while organophosphorus yolk medium is targeted at evaluating organic phosphorus-degrading capacity, and a halo zone on either medium indicates that the bacterium can break down the corresponding phosphorus compound. Uninoculated blank media were included as negative controls, which showed no halo zones under the same conditions. We found that TCX1 formed a clear halo zone around its colonies on PKO solid medium, but not on organophosphorus yolk solid medium (Figure 1A,C). The halo on PKO medium had an E/e ratio (where E is the diameter of the phosphate-solubilizing halo and e is the diameter of the colony) of 1.04 ± 0.02, confirming TCX1’s specific capacity to solubilize inorganic phosphorus (Figure 1C). Furthermore, TCX1 was found to produce ACC deaminase with activity ranging from 0.55 to 0.59 μM·mg^−1^·h^−1^. Similar halo zones were also observed on Chrome Azurol S (CAS) medium, indicating that TCX1 produced siderophores that bind insoluble ferric ions, which in turn facilitates plant iron acquisition through chelate degradation or ligand exchange in root systems (Figure 1E). Therefore, the antagonistic strain TCX1 possesses significant plant growth-promoting functions.

### 2.3. Bacillus subtilis Strain TCX1 Demonstrated Stable Colonization in Cucumber Seedlings

The colonization ability of the functional strain TCX1 is a prerequisite for its disease control efficacy, as it directly determines the strain’s capacity to exert beneficial effects. To assess whether TCX1 can colonize cucumber seedlings and thereby support subsequent resistance against *FOC*, we employed a rifampicin-based colonization tracking approach. This common strategy requires a resistant derivative of the target strain to allow unambiguous identification and re-isolation from plant tissues. We therefore generated a rifampicin-resistant variant of TCX1, capable of growing stably at 300 μg/mL rifampicin, which we designated Rif-TCX1. This marked strain was then applied to cucumber seedlings.

**Figure 2:**
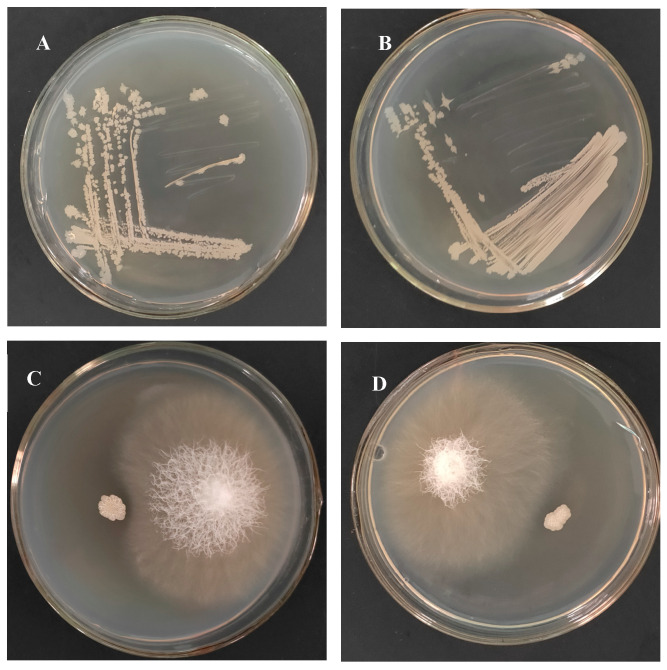
Comparison of morphology and antagonism between original TCX1 and Rif-TCX1 towards *FOC*. (**A**) Original TCX1 colonies; (**B**) Rif-TCX1 colonies; (**C**) TCX1 against *FOC*; (**D**) Rif-TCX1 against *FOC*. The experiments were repeated three times, and representative images are shown.

After 21 d of cultivation, Rif-TCX1 colonies were successfully re-isolated from the inoculated plants. Colony morphology comparison showed no differences in color or shape between Rif-TCX1 and the wild-type TCX1 (Figure 2A,B). In addition, a plate confrontation assay confirmed that the antagonistic activity of Rif-TCX1 against *FOC* remained unchanged compared to the original strain (Figure 2C,D). Rif-TCX1 was recovered from the roots, stems, and leaves of inoculated seedlings, whereas no rifampicin-resistant colonies were detected in control plants. These results demonstrate that TCX1 possesses strong colonization ability in cucumber seedlings, which is critical for its role in promoting plant growth and conferring resistance to *FOC*.

### 2.4. Enhanced Biocontrol of Cucumber Fusarium Wilt by Bacillus subtilis Strain TCX1

**Figure 3:**
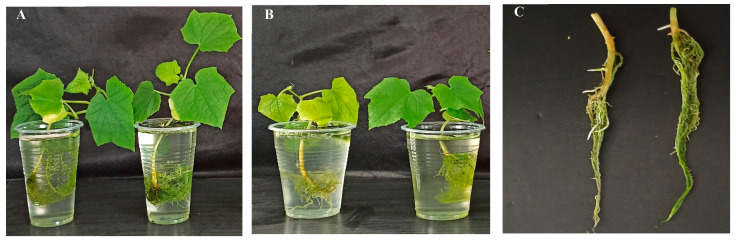
Growth promotion and biocontrol effects of TCX1 on cucumber seedlings. (**A**) Cucumber seedlings treated with sterile culture medium (control, left) and treated with TCX1 (right); (**B**) Cucumber seedlings inoculated with *FOC* alone (left) and pretreated with TCX1 followed by *FOC* (right); (**C**) Roots of cucumber seedlings inoculated with *FOC* alone (left) and pretreated with TCX1 prior to *FOC* inoculation (right). Each treatment group included three biological replicates, with five uniform 30-day-old cucumber seedlings per replicate, resulting in a total of 15 plants per group. The entire experiment was independently repeated three times. Representative images are shown.

In greenhouse pot experiments, cucumber seedlings inoculated with strain TCX1 (right side of Figure 3A) exhibited significant increases in height, fresh weight, and dry weight compared to the non-inoculated control (left side of Figure 3A), demonstrating the strain’s substantial growth-promoting effect (Figure 3A). Seedlings inoculated with *FOC* alone (left sides of Figure 3B,C) showed clear wilting and yellowing of leaves, stems, and roots. In contrast, these symptoms were greatly alleviated in seedlings that had been pre-inoculated with TCX1 before *FOC* challenge (right sides of Figure 3B,C). Statistical analysis further supported these observations: the disease index reached 69.8% in the *FOC*-only treatment but was reduced to 40.5% in the TCX1 + *FOC* co-treatment. This represents a 41.9% reduction in the disease index, confirming the effectiveness of TCX1 in mitigating cucumber *Fusarium* wilt.

### 2.5. Damaging Activity of Bacillus subtilis Strain TCX1 Against FOC

#### 2.5.1. *Bacillus subtilis* Strain TCX1 Exhibits Hydrolase Activity and High Likelihood of Producing Lipopeptides

**Figure 4:**
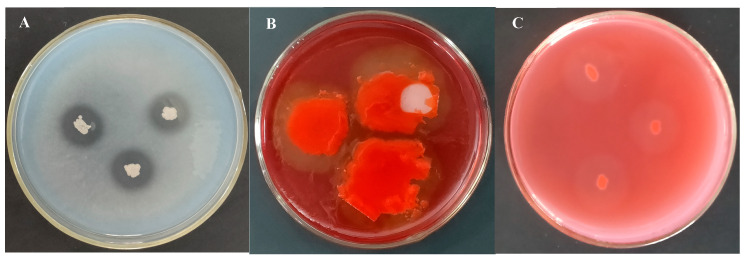
Enzyme-producing activity of TCX1 during metabolism. (**A**) Strain TCX1 was incubated at 30 °C in the dark for 2 d to detect its protease-producing activity; (**B**) Strain TCX1 was incubated at 30 °C in the dark for 2 d, after which the plate was stained by soaking in a red dye solution for 2 h; the dye solution was then discarded, and 1 M NaCl solution was added for a 3 h soak to detect its cellulase-producing activity; (**C**) Strain TCX1 was incubated at 30 °C in the dark for 2 d to detect its β-glucanase-producing activity. The experiments were repeated three times, and representative images are shown.

To determine how *Bacillus subtilis* strain TCX1 enhances cucumber tolerance to *FOC*, we used TCX1 DNA as a template and primers from Table S2 to amplify gene fragments related to biosynthesis of lipopeptides via PCR. The results showed that the strain encodes three lipopeptide-encoding genes and thus is highly likely to metabolize and produce three types of lipopeptides: surfactin, iturin, and fengycin. The cell wall of *FOC* is composed of cellulose, β-glucan, and a small amount of protein. To assess the hydrolytic activity of TCX1, the strain was cultured on substrate-supplemented media, using uninoculated media as blank controls. Clear zones were observed around TCX1 colonies on the respective media, confirming that TCX1 secretes protease, cellulase, and β-glucanase during its growth (Figure 4A–C). These hydrolytic enzymes may directly damage the cell wall of *FOC*. Additionally, TCX1 was found to produce siderophores, which may inhibit the growth of phytopathogens by depleting iron in the environment (Figure 1C).

#### 2.5.2. *Bacillus subtilis* Strain TCX1 Impaired the Cell Membrane of *FOC*

**Figure 5:**
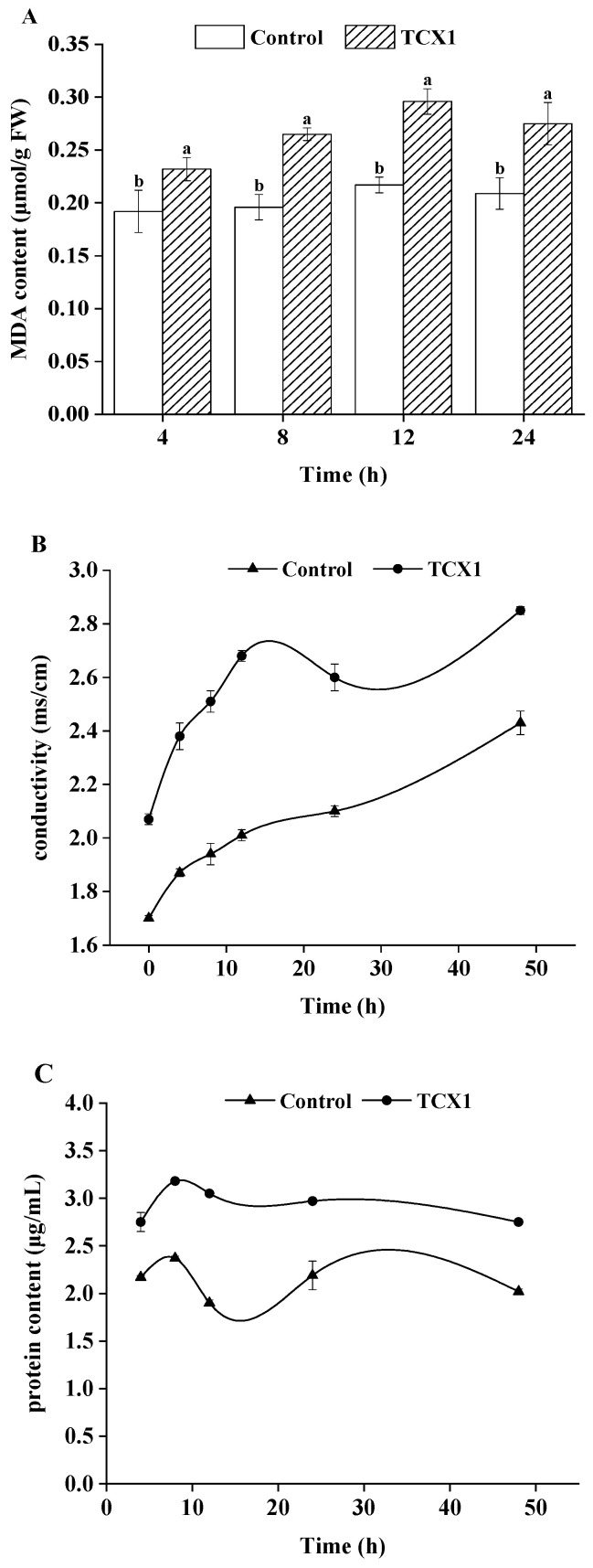
Effect of co-culture of *Bacillus subtilis* strain TCX1 with *FOC* on *FOC* mycelia membrane stability. (**A**) 10 mL of TCX1 (OD_600_ = 1.0) was added to *FOC* cultures, with an equal volume of sterile water as control. Samples were analyzed at 4 h, 8 h, 12 h, and 24 h post-treatment; *FOC* mycelial malondialdehyde (MDA) content; (**B**) Electrical conductivity of the mixture; (**C**) *FOC* intracellular protein leakage. Each treatment had three biological replicates, and the entire experiment was repeated three times independently. A significant difference between the TCX1-treated group and the control group is denoted by different letters (*p* < 0.05).

Based on the finding that TCX1 produces a range of hydrolases and lipopeptides, we hypothesized that it could disrupt the cell membrane integrity of *FOC*. To test this, we assessed the extent of membrane damage after co-cultivation with TCX1 by quantifying malondialdehyde (MDA) content (an indicator of lipid peroxidation), measuring cell membrane permeability, and detecting intracellular protein leakage—over the entire treatment period (0–24 h), these three indicators in the TCX1-treated group were significantly higher than those in the control group (Figure 5A–C). Our results showed that TCX1 induced lipid peroxidation in the *FOC* membranes, as evidenced by a progressive increase in MDA content. This peroxidative damage was accompanied by increased membrane permeability, which led to significant leakage of intracellular proteins.

#### 2.5.3. *Bacillus subtilis* Strain TCX1 Impeded the Growth of *FOC* Mycelium and the Germination of *FOC* Spores

**Figure 6:**
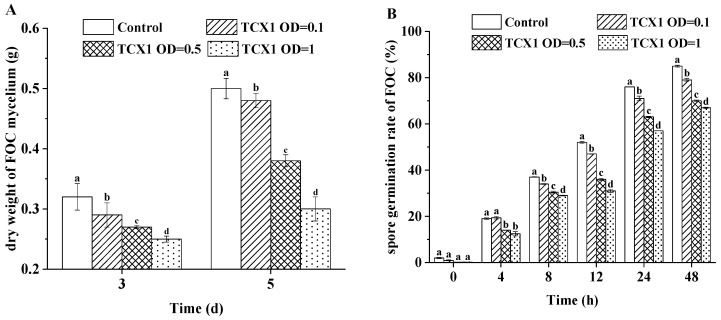
Effect of TCX1 on *FOC* mycelial growth and spore germination. (**A**) *FOC* and various concentrations of TCX1 were co-incubated at 120 rpm and 25 °C for durations of 3 d and 5 d. The dry weight of the *FOC* mycelium was measured; (**B**) *FOC* and TCX1 were co-incubated under the same conditions. The germination of spores was observed under a microscope at time points of 0, 4, 8, 12, 24, and 48 h, and the germination rate was calculated accordingly. Each treatment was conducted with three replicates, and the entire experiment was repeated three times independently. Significant differences between different treatments are denoted by different letters (*p* < 0.05).

Furthermore, we co-cultured *FOC* with various concentrations of TCX1 and measured mycelial growth at 3 d and 5 d, as well as spore germination rates at 0, 4, 8, 12, 24, and 48 h. Our findings showed that in the control group, mycelial growth increased significantly between day 3 and day 5, indicating rapid proliferation in the absence of TCX1. Conversely, TCX1 treatment resulted in a dose-dependent reduction in mycelial growth, which was consistently lower than that of the control at all concentrations tested (Figure 6A). Furthermore, a similar dose-dependent inhibitory effect was observed on spore germination (Figure 6B). Therefore, TCX1 effectively suppressed both *FOC* mycelial growth and spore germination in a concentration-dependent manner.

#### 2.5.4. FOC Upregulated Metabolic Enzymes to Counteract the Detrimental Effects of *Bacillus subtilis* strain TCX1

**Figure 7:**
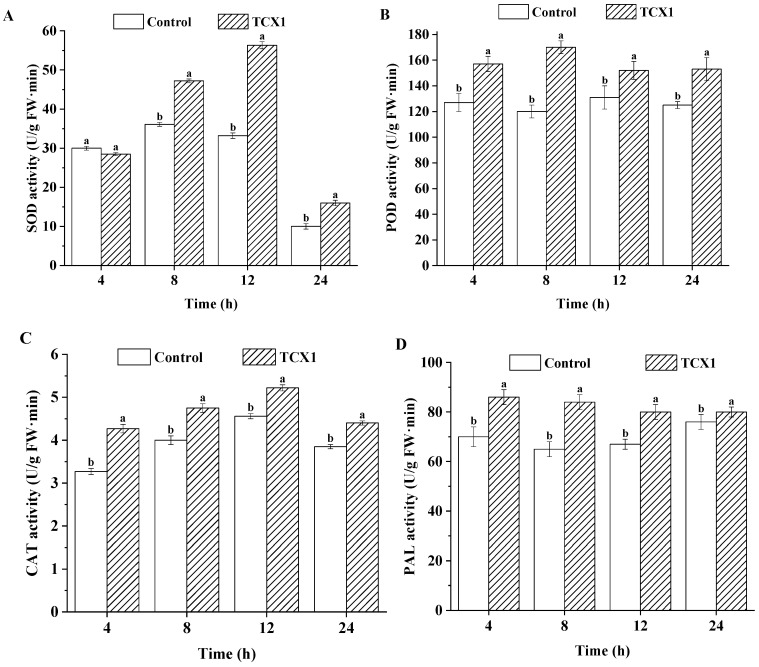
The effect of TCX1 on *FOC* metabolic enzymes. (**A**) *FOC* was cultivated in 80 mL of potato dextrose broth (PDB) at 120 rpm, 25 °C for 3 d. Then, 10 mL of TCX1 solution (OD_600_ = 1.0) was added to each group, with an equal volume of sterile water as the control. The SOD activity in *FOC* was assessed at 4 h, 8 h, 12 h, and 24 h post-treatment. (**B**) POD activity. (**C**) CAT activity. (**D**) PAL activity. Each treatment was conducted with three replicates, and the entire experiment was repeated three times independently. Significant differences between different treatments are denoted by different letters (*p* < 0.05).

To counteract the stress induced by TCX1, *FOC* modulated its enzymatic defense systems. We compared the activities of superoxide dismutase (SOD), catalase (CAT), peroxidase (POD), and phenylalanine ammonia-lyase (PAL) in *FOC* between a control group and a treatment group co-cultured with TCX1. In the control group, SOD activity remained stable within the first 12 h, increased to a peak, and subsequently declined. The TCX1-treated group showed a similar trend but exhibited a more pronounced increase in SOD activity, reaching a significantly higher maximum, suggesting an enhanced oxidative stress response to TCX1 (Figure 7A). POD activity remained stable in the control but increased rapidly in the treated group, stabilizing at a level significantly higher than the control (Figure 7B). CAT activity followed a similar trend in both groups but was consistently and significantly higher in the TCX1-treated group (Figure 7C). PAL activity in the treated group rose rapidly to a peak and maintained a stable level significantly above that of the control (Figure 7D). In conclusion, when challenged by the antagonist *Bacillus subtilis* strain TCX1, *FOC* upregulates key antioxidant and defense-related enzymes to alleviate the resulting damage.

### 2.6. Bacillus subtilis Strain TCX1 Induces Cucumber Tolerance to FOC

#### 2.6.1. *Bacillus subtilis* Strain TCX1 Improved Plant Antioxidants Expression in Cucumber

**Figure 8:**
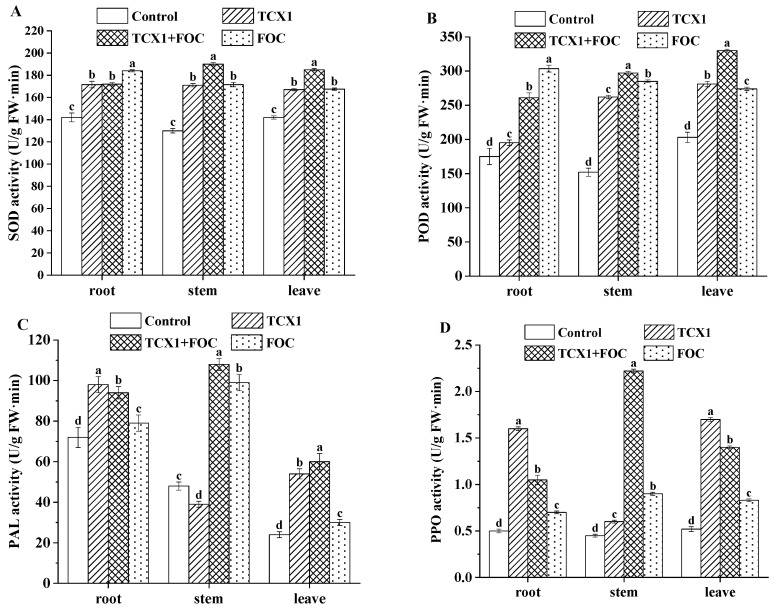
TCX1 enhances the activities of antioxidant enzymes in cucumber seedlings. (**A**) Cucumber seedlings were treated in four groups as follows: (1) control, without any microbial treatment; (2) TCX1, inoculation with strain TCX1; (3) TCX1 + *FOC*, co-inoculation with strain TCX1 and *FOC*; (4) *FOC*, inoculation with *FOC*. For enzyme activity determination, 0.5 g of fresh sample from each treatment was placed in a mortar, and 5.0 mL of enzyme extraction buffer was added. The mixture was ground into a homogenate under ice bath conditions and centrifuged at 4 °C and 10,000 rpm for 30 min. The supernatant was collected to measure SOD activity; (**B**) POD activity; (**C**) PAL activity; (**D**) PPO activity. Each treatment was conducted with three replicates, and the entire experiment was repeated three times independently. Significant differences between different treatments are denoted by different letters (*p* < 0.05).

Beyond directly combating *FOC*, TCX1 also induced indirect resistance in cucumbers in cucumber plants. When cucumbers were infected with *FOC*, the activities of antioxidant enzymes—including SOD, POD, PAL, and PPO—were upregulated in the roots, stems, and leaves (Figure 8). Notably, TCX1 alone also elicited a similar upregulating effect. Even before *FOC* infection, the activities of SOD, POD, PAL, and PPO in all organs of cucumber seedlings were already induced and enhanced by TCX1. This pre-activation of the plant defense system established a preemptive mechanism that mitigated subsequent damage caused by *FOC* infection.

#### 2.6.2. *Bacillus subtilis* Strain TCX1 Triggered the Induction of ISR and SAR in Cucumber

**Figure 9:**
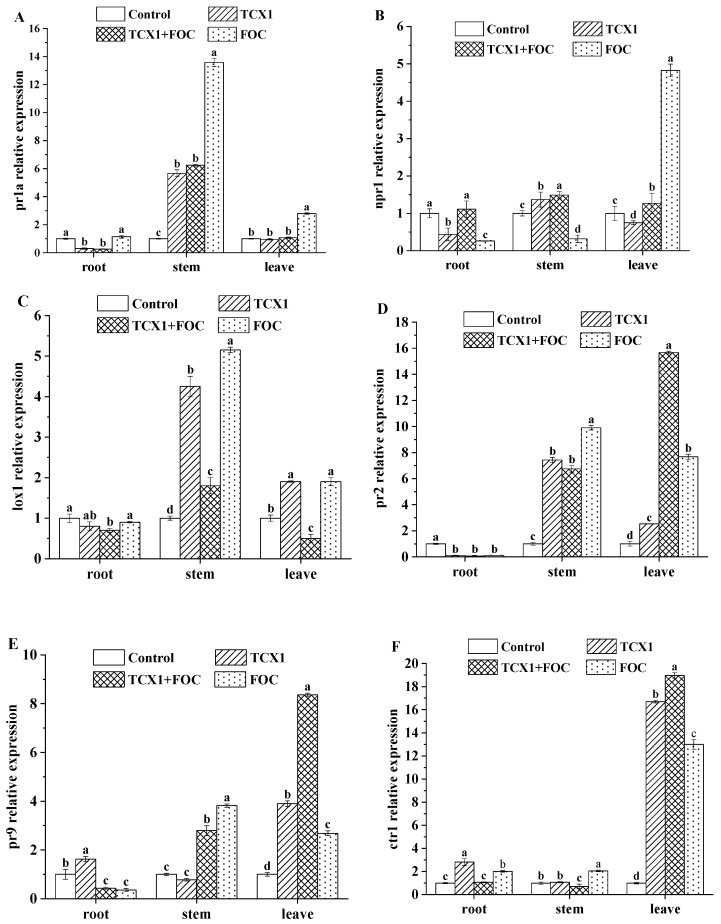
Effects of TCX1 on the expression of ISR and SAR marker genes in different organs of cucumber seedlings. (**A**) Cucumber seedlings were treated in four groups as follows: (1) control, without any microbial treatment; (2) TCX1, inoculation with strain TCX1; (3) TCX1 + *FOC*, co-inoculation with strain TCX1 and *FOC*; (4) *FOC*, inoculation with the pathogenic fungus *FOC*. qRT-PCR quantification of *pr1a* transcript levels in roots, stems, and leaves; (**B**) *npr1* transcript levels; (**C**) *lox1* transcript levels; (**D**) *pr2* transcript levels; (**E**) *pr9* transcript levels; (**F**) *ctr1* transcript levels. Each treatment was conducted with three replicates, and the entire experiment was repeated three times independently. Significant differences between different treatments are denoted by different letters (*p* < 0.05).

The genes *npr1*, *pr2*, *pr1a*, *lox1*, *pr9*, and *ctr1* are key markers for induced systemic resistance (ISR) and systemic acquired resistance (SAR). Among them, *pr1a* and *npr1* are canonical markers of the salicylic acid (SA) pathway; *lox1* is a key marker of the jasmonic acid (JA) pathway; *pr2* is primarily involved in the SA pathway; *pr9* expression is regulated by the SA, JA, and ethylene (ET) pathways; and *ctr1* participates in the ET pathway. To determine whether *Bacillus subtilis* strain TCX1 triggers ISR and SAR in cucumber, we performed qRT-PCR to analyze the expression of these genes in seedlings subjected to four treatments: control (no microbial treatment), TCX1 (inoculated with TCX1), *FOC* (inoculated with *FOC*), and TCX1 + *FOC* (co-inoculated with TCX1 and *FOC*). Following *FOC* inoculation, the expression of all six genes—*pr1a*, *npr1*, *lox1*, *pr2*, *pr9*, and *ctr1*—was upregulated in nearly all tissues, indicating that cucumber activates both ISR and SAR pathways as a defense mechanism against *Fusarium* wilt. In cucumbers inoculated with TCX1 alone, *pr1a* and *npr1* expression was upregulated in the stems (Figure 9A,B). Additionally, *lox1* and *pr2* showed significant upregulation in both stems and leaves (Figure 9C,D), while *pr9* and *ctr1* were primarily upregulated in the roots and leaves (Figure 9E,F). These findings suggest that *Bacillus subtilis* strain TCX1 activates distinct ISR- and SAR-related pathways in different cucumber organs, thereby enhancing the plant’s systemic defense against cucumber *Fusarium* wilt.

## 3. Discussion

Cucumber, an important cash crop, is highly susceptible to *Fusarium oxysporum* f. sp. *Cucumerinum* (*FOC*) during cultivation, making its control a crucial concern for improving yield [7]. Endophytes with antipathogenic activities provide sustainable agricultural strategies to mitigate abiotic and biotic stresses, promoting plant growth and enhancing disease resistance [12,29,30]. In this study, we isolated 14 disease-resistant endophytic bacterial strains from *Ambrosia artemisiifolia*, identifying them based on morphological characteristics and 16S rDNA sequencing. Among these, we identified a functional strain, *Bacillus subtilis* strain TCX1, which through comprehensive physiological, biochemical, and genetic analyses, demonstrated significant plant growth-promoting capabilities and potent antagonistic effects against cucumber *Fusarium* wilt.

Plant endophytes enhance growth by producing phytohormones, particularly IAA [31,32]. Previous studies have highlighted the critical role of IAA and ACC deaminase in the plant growth-promoting activities of *Bacillus subtilis* [33]. In our study, TCX1 colonization of cucumber plants was associated with the synthesis of IAA and production of ACC deaminase, leading to promoted growth. These findings are consistent with reports on *Bacillus subtilis* KU21 isolated from *Rosmarinus officinalis* roots, which enhanced tomato survival [34]. Additionally, IAA has been shown to significantly improve chilling tolerance in cucumber seedlings by modulating H_2_S-mediated responses [9]. We also found that TCX1 solubilizes inorganic phosphorus and secretes siderophores, thereby improving phosphorus uptake and synergistically promoting plant growth. Schmidt et al. reported that endophytes exhibiting strong siderophore production and phosphorus solubilization in vitro were the most effective at promoting growth under phosphorus-limiting conditions in pot experiments [35]. Similarly, Jensen et al. demonstrated that *Bacillus subtilis* strain ALC_02 facilitates plant phosphorus (P) acquisition through P solubilization and stimulation of root and root hair development [36]. Furthermore, *Bacillus subtilis* can act synergistically with other strains, such as *Bacillus megaterium* CNPMS B119 and *Bacillus subtilis* CNPMS B2084, to improve phosphorus acquisition and maize yield [37]. Therefore, our results confirm that inoculation with the endophytic bacterium TCX1 significantly promotes the growth of cucumber plants.

The disease resistance mechanisms of *Bacillus subtilis* typically include disruption of pathogen cell membranes, production of antimicrobial compounds, and induction of systemic resistance in plants [21,38,39]. Our investigation into TCX1’s antagonistic capabilities revealed that it produces cell wall-degrading enzymes—including protease, cellulase, and β-glucanase—and is highly likely to produce lipopeptides, both of which contribute directly to the disruption of *FOC* mycelia membranes. To further validate this lipopeptide production, LC-MS/MS quantitative analysis will be conducted as a key priority in subsequent research. Romero et al. also reported that lipopeptides not only directly suppress pathogenic fungi but also induce systemic resistance in host plants [40]. Furthermore, when co-cultured with TCX1, both the germination rate of *FOC* spores and mycelial growth were significantly inhibited, consistent with previous studies by Gao et al. and Yi et al. [41,42].

Additionally, *Bacillus subtilis* strain TCX1 enhanced *FOC* tolerance in cucumber by upregulating antioxidant production and inducing both ISR and SAR. Colonization by TCX1 increased the activity of antioxidant enzymes in cucumbers. Peroxidase (POD), a key enzyme in intracellular reactive oxygen species metabolism in *Bacillus subtilis*, showed enhanced activity. Similarly, inoculation of tomatoes with *Bacillus subtilis* SR22 increased POD activity by 56%, improving defense against *Rhizoctonia* root rot [43]. Phenylalanine ammonia-lyase (PAL), a pivotal enzyme in the biosynthesis of secondary metabolites, is closely correlated with plant defense mechanisms against pathogens. Superoxide dismutase (SOD), a detoxifying enzyme, effectively scavenges reactive oxygen species (ROS), protecting cells from oxidative damage [44]. Thus, TCX1 enhanced cucumber resistance by boosting the activity and expression of key antioxidant enzymes.

Studies have shown that *Bacillus subtilis* can elicit ISR and SAR by inducing the accumulation of regulatory genes such as *npr1* and various pathogenesis-related (*pr*) genes, thereby enhancing the plant’s disease resistance. The reduction in disease incidence mediated by ISR has been documented in diverse plant species, including tomato, cabbage, and lettuce [45,46]. Although SAR typically has a narrower induction spectrum than ISR, it has been reported that *Bacillus subtilis* strain 47, combined with chitosan and its derivatives, can elicit SAR in potato plants against viral diseases [47]. In our study, we observed upregulation of ISR- and SAR-related genes—*pr1a*, *npr1*, *lox1*, *pr2*, *pr9*, and *ctr1*—across various cucumber tissues. These findings collectively indicate that TCX1 enhances cucumber resistance against *Fusarium* wilt by modulating these defense pathways. Nevertheless, research on the regulation of ISR and SAR remains at the transcriptional level. Confirming these mechanisms at the hormonal level will therefore require the precise quantification of phytohormones via LC-MS/MS in future studies.

When TCX1 colonized cucumber seedlings prior to *FOC* infection, it conferred superior resistance compared to simultaneous inoculation with *FOC*. This suggests that, beyond direct antagonism, TCX1 may also control *Fusarium* wilt by competing with *FOC* for space and nutrients. To facilitate the application of TCX1, we optimized its culture conditions using response surface methodology (RSM), based on preliminary one-way experiments. The optimal fermentation conditions were identified as follows: nitrogen source 11 g/L, carbon source 16 g/L, and initial pH 7.0. Given its efficacy and environmental friendliness, TCX1 shows considerable potential as a biocontrol agent against *FOC*, offering a promising solution for managing cucumber *Fusarium* wil. Given its efficacy and environmental compatibility, TCX1 demonstrates significant potential as a biocontrol agent against *FOC*. To harness this potential for managing cucumber *Fusarium* wilt, field experiments and multi-season trials will be crucial in future work.

In conclusion, we have demonstrated the pivotal role of *Bacillus subtilis* strain TCX1 in promoting cucumber growth and enhancing resistance to *Fusarium* wilt caused by *FOC*. Nevertheless, several aspects remain unresolved, including the precise mechanisms of TCX1-induced ISR and SAR, the regulation of these pathways, and other potential modes of action against *FOC*. To fully exploit TCX1’s potential, further research is warranted, including genome sequencing and analysis, screening for active antimicrobial compounds, and transcriptomic profiling. Additionally, given TCX1’s broad-spectrum activity against various plant pathogens—as evidenced by its significant inhibition rates against *Fusarium graminearum* (66.9%), *Sclerotinia sclerotiorum* (77.0%), and *Fusarium oxysporum* f. sp. *Melonis* (71.7%) (Table 2)—future studies should explore its application against other pathogens and elucidate the underlying resistance mechanisms.

## 4. Materials and Methods

### 4.1. Endophytic Bacteria Isolation

Isolation of bacterial endophytes was conducted from the roots, stems, and leaves of ragweed collected from Shenyang Normal University in Shenyang City, Liaoning Province, China (41°54′ N, 123°24′ E, 41.45 m above sea level). Healthy plants were plucked, placed in sealed bags, and transported to the laboratory in an icebox. Within 12 h of collection, the plants were rinsed with sterile water for 30 min, dried, and cut into small pieces (3 mm) in a sterile environment. Endophytic bacteria were isolated and purified [48]. The bacteria were streaked onto nutrient agar (NA) medium (per liter of distilled water: beef extract 3.0 g, peptone 10 g, NaCl 5 g, and agar 20.0 g, pH 7.0–7.2), incubated at 30 °C for 48 h, and observed for morphological characteristics. Purified bacteria were preserved in 50% (*v*/*v*) glycerol at −80 °C for future use.

### 4.2. Identification of the Endophytic Bacteria by 16S rRNA Sequencing

Genomic DNA was extracted and the 16S rRNA gene was amplified using the universal primers 27F (5′-AGAGTTTGATCCTGGCTCAG-3′) and 1492R (5′-GGTTACCTTGTTACGACTT-3′). The PCR products were purified and sequenced by Sangon Biotech (Shanghai, China). The resulting sequences were analyzed using the BLAST+ 2.9.0 program on the NCBI database. Sequences showing >98% identity to known bacterial 16S rDNA sequences were selected for further analysis [49].

### 4.3. Plate Confrontation Method

Twelve preserved strains of pathogenic fungi were first inoculated onto potato dextrose agar (PDA) medium (composition per liter: 200 g potato, 20 g glucose, and 18.0 g agar) and activated by incubation at 28 °C for 3 to 4 d. Using a sterile cork borer with a diameter of 5 mm, fungal plugs were aseptically obtained from the edge of the freshly grown mycelium of each strain and inoculated at the center of fresh PDA plates. For the initial screening, isolated endophytic bacterial strains were inoculated at 1.5 cm away from the center along two perpendicular radii. Each plate accommodated the inoculation of four endophytic bacterial strains. Strains exhibiting antifungal activity were selected for subsequent analysis. In the subsequent confirmation assay, fungal plugs were inoculated on the opposite side of the PDA plate relative to the candidate endophytic bacterial strains, maintaining a distance of 3.5 cm between them. PDA plates inoculated with the pathogenic fungus alone served as the negative controls. The experiment was conducted with three independent biological replicates, and all plates were incubated at 28 °C. The inhibitory effects of the endophytic bacterial strains on the radial growth of the pathogenic fungi were observed and recorded. The antifungal inhibition rate was calculated as follows: K (%) = [(R__control_ − r__treatment_)/R__control_] × 100%, where R__control_ represents the colony radius of pathogenic fungi in the experimental control group (in cm) and r__treatment_ denotes the colony radius of pathogenic fungi in the experimental treatment group (in cm).

### 4.4. Biological Characteristics of the Strain

#### 4.4.1. Phosphate Solubilization

After activation, the screened antagonistic strains were inoculated into 50 mL of PDB medium (per liter of distilled water: potato infusion 4.0 g and glucose 20 g) and cultured at 37 °C with shaking at 180 rpm for 24 h. Subsequently, 2.5 μL of the bacterial suspension was pipetted onto solid plates of Pikovskaya (PKO) medium (per liter of distilled water: glucose 10 g, (NH_4_)_2_SO_4_ 0.5 g, NaCl 0.3 g, KCl 0.3 g, MgSO_4_·7H_2_O 0.3 g, MnSO_4_·H_2_O 0.03 g, FeSO_4_·7H_2_O 0.03 g, Ca_3_(PO_4_)_2_ 2.5 g, and agar 18.0 g, pH 7.0–7.5), and the plates were incubated at 37 °C for 7 d. The blank medium served as a negative control. For the assessment of dissolved organic phosphorus, the PKO solid plates were replaced with organophosphorus yolk solid medium (glucose 10 g, (NH_4_)_2_SO_4_ 0.5 g, NaCl 0.3 g, KCl 0.3 g, MgSO_4_·7H_2_O 0.3 g, MnSO_4_·H_2_O 0.03 g, FeSO_4_·7H_2_O 0.03 g, CaCO_3_ 5 g, sterile egg yolk 50 mL, agar 18.0 g, distilled water 1000 mL, pH 7.0~7.5), while all other operations remained the same as those for the inorganic phosphorus test. The diameters of both the halo zone and the colony were measured using digital calipers. The phosphate solubilizing activity was evaluated using the phosphorus solubilizing index (SI), calculated as follows: SI = E/e (diameter of halo zone/colony diameter) [50]. The SI values were then recorded.

#### 4.4.2. Indole Acetic Acid (IAA) Production

King’s B liquid medium (per liter: peptone 20 g, glycerol 15 mL, K_2_HPO_4_ 1.5 g, MgSO_4_·7H_2_O 1.5 g, pH 7.2) supplemented with 0.05% (*w*/*v*) L-tryptophan was inoculated with 1% (*v*/*v*) of an activated culture of strain TCX1 and incubated at 37 °C with shaking at 180 rpm for 2 d. For qualitative detection of IAA, a 100 μL aliquot of the bacterial culture was pipetted onto a white ceramic plate, and an equal volume of freshly prepared Salkowski colorimetric reagent (1 mL of 0.5 mol/L FeCl_3_ solution dissolved in 50 mL of distilled water; 30 mL of concentrated sulfuric acid was added, followed by dilution to 100 mL with distilled water) was added. The mixture was reacted in the dark for 15 min. Blank medium was used as the negative control, and 100 μg/mL IAA standard solution was used as the positive control; the formation of a pink color was observed. For quantitative determination of IAA, 5 mL of supernatant was collected and mixed with 5 mL of Salkowski colorimetric reagent. After reaction in the dark for 30 min, the absorbance of the mixture was measured at 530 nm. The IAA concentration was calculated using a standard curve constructed with IAA standards. All tests were performed in triplicate to ensure consistency.

#### 4.4.3. ACC Deaminase Activity

The test strain was activated, inoculated into PDB medium, and cultured at 30 °C with shaking at 180 rpm for 24 h. After centrifugation, the bacterial pellet was rinsed three times with sterile DF minimal salt medium and resuspended in ADF induction medium. The composition of the DF and ADF media (per liter) was as follows: KH_2_PO_4_ 4 g, Na_2_HPO_4_ 6 g, MgSO_4_·7H_2_O 0.2 g, Glucose 2 g, Gluconic acid 2 g, Citric acid 2 g; DF was supplemented with (NH_4_)_2_SO_4_ 2 g as the nitrogen source, while ADF was supplemented with 3.0 mmol/L ACC as the sole nitrogen source. Both media were supplemented with 1 mL/L of a filter-sterilized 1000× trace elements solution (H_3_BO_3_ 10 g, MnSO_4_·H_2_O 11.4 g, ZnSO_4_·7H_2_O 77.8 g, CuSO_4_·5H_2_O 7.8 g, Na_2_MoO_4_·2H_2_O 10 g, dissolved in 1 L distilled water). The resuspended bacteria in ADF medium were incubated again at 30 °C with shaking at 180 rpm for 24 h. The cells were harvested by centrifugation, and the pellet was resuspended in 0.6 mL of 0.1 M Tris-HCl buffer (pH 8.5). A 200 μL aliquot of this bacterial suspension was mixed with 20 μL of 0.5 M ACC solution (test group), and another 200 μL aliquot was mixed with 20 μL of sterile water (negative control). The reaction proceeded at 30 °C for 15 min and was then terminated by adding 1 mL of 0.56 M HCl. After centrifugation at 11,000× *g* for 5 min at room temperature, 1 mL of the supernatant was transferred to a new tube and mixed with 1 mL of 0.56 M HCl. Then, 300 μL of 0.2% (*w*/*v*) 2,4-dinitrophenylhydrazine reagent (in 2 M HCl) was added, and the solution was incubated at 30 °C for 30 min. Finally, 2 mL of 2 M NaOH was added, and the mixture was vortexed thoroughly. The absorbance of the resulting solution was measured at 540 nm using a spectrophotometer (Model T-UV1810, Shanghai Youke Instrument Co., Shanghai, China). The α-ketobutyrate concentration produced from the hydrolysis of ACC was calculated from a standard curve prepared with known concentrations of α-ketobutyrate and was used to represent the ACC deaminase activity.

#### 4.4.4. Enzyme Activity Assay of Endophytic Bacteria

Protease activity assay: TCX1 was inoculated into protease assay medium (NA medium supplemented with 1% skim milk powder, pH 7.0~7.2) and incubated in the dark at 30 °C for 2 d. The presence or absence of a transparent circle was then observed. The experiments were repeated three times.

Cellulase activity assay: TCX1 was inoculated into Carboxymethylcellulose Sodium (CMC) medium (per liter of distilled water: peptone 10 g, KH_2_PO_4_ 1 g, NaCl 5 g, yeast extract powder 10 g and Sodium Carboxymethylcellulose 10 g, pH 7.0~7.2) and cultured in the dark at 30 °C for 2 d. The plates were immersed in Congo red dye for 2 h for staining, followed by pouring off the dye. Subsequently, 1 M NaCl solution was added for immersion for 3 h. The presence or absence of a transparent circle was then observed. The experiments were repeated three times.

β-glucanase activity: TCX1 was cultured in a β-glucanase identification medium (per liter of distilled water: β-glucan 2 g, NaNO_3_ 2 g, KH_2_PO_4_ 1 g, KCl 0.5 g, MgSO_4_ 0.5 g, FeSO_4_ 0.01 g, Congo Red 0.05 g and agar 18 g, pH 7.0~7.2) at 30 °C for 2 d. The presence or absence of a transparent circle was then observed. The experiments were repeated three times.

Siderophore activity: Chrome Azurol S (CAS) medium (per liter of distilled water: CAS 60.5 mg, 1 mmol/L FeCl_3_ 50 mL, Cetyltrimethylammonium Bromide 72.9 mg, Glucose 9 g, beef extract powder 2.7 g, Peptone 4.5 g, NaCl 5 g and agar 18 g, pH 7.0~7.2) was used for detection. TCX1 was cultured at 30 °C for 4 d to observe the formation of orange-yellow halos. The experiments were repeated three times.

### 4.5. Assessment of Colonization Ability of Bacillus subtilis TCX1

The colonization ability of *Bacillus subtilis* TCX1 in cucumber seedlings was determined using a standard methodology [51]. TCX1 was activated and grown in NA media with rifampicin concentrations ranging from 20 to 300 μg/mL at 30 °C with shaking at 160 rpm for 24 h. Strains resistant to 300 μg/mL rifampicin (Rif-TCX1) were selected and passaged for five generations to ensure stability. A single Rif-TCX1 colony was isolated by streaking, and its colony morphology and disease resistance were confirmed to be consistent with the wild-type strain before use. Cucumber seedlings were inoculated with Rif-TCX1 and cultivated for 21 d. Rif-TCX1 was then isolated from cucumber tissue, and its colonization within the plants was confirmed using plates containing 300 μg/mL rifampicin.

### 4.6. Greenhouse Pot Experiment

Greenhouse pot experiments were conducted to evaluate the biocontrol efficacy of *Bacillus subtilis* TCX1 against *Fusarium* wilt of cucumber. Surface-sterilized cucumber seeds were germinated and grown until the two-leaf stage. The seedlings were then treated with the antagonistic strain TCX1 at OD_600_ = 1.0. After 2–3 d, they were challenged with *FOC.* The experiment was arranged in a randomized complete block design with the following four treatments (each with three replicates): (1) Control (no microbial treatment), (2) TCX1 (inoculation with *Bacillus subtilis* TCX1), (3) *FOC* (inoculation with *FOC*), and (4) TCX1 + *FOC* (co-inoculation with *Bacillus subtilis* TCX1 and *FOC*). Each replicate consisted of five uniform 30-day-old cucumber seedlings, resulting in a total of 15 plants per treatment group. The entire experiment was independently repeated three times. The seedlings’ disease development and growth status were regularly monitored. Disease incidence (DI) was calculated using the formula: DI = Σ (Number of diseased plants × Disease grade)/(Highest disease grade × Total number of plants) × 100. Control efficiency (%) = [(DI__control_ − DI__treatment)_/DI__control_] × 100. The disease severity scale for cucumber seedlings was defined as follows: Grade 0 (healthy, no symptoms), Grade 1 (<1/4 vascular tissues yellow, ≤50% leaves wilted), Grade 2 (1/2 vascular tissues yellow, >50% leaves wilted), Grade 3 (3/4 vascular tissues yellow, leaves withering, only growing point alive), and Grade 4 (>3/4 vascular tissues yellow or plant death).

### 4.7. FOC Mycelium Growth and Spore Germination Assays

#### 4.7.1. Mycelial Growth Inhibition Assay

The TCX1 culture was diluted to OD_600_ values of 0.1, 0.5, and 1.0, and added to PDB medium containing 20 mL of *FOC* culture. Sterile water was added as a control in equal volume. The mixtures were co-incubated at 120 rpm and 25 °C for 3–5 d. After incubation, the entire culture was harvested by filtration through pre-dried and weighed filter paper. The mycelia were washed thoroughly with distilled water and dried at 80 °C to a constant weight. The dry weight of *FOC* mycelium was determined by calculating the weight difference. Each treatment consisted of three biological replicates, and the entire experiment was independently repeated three times.

#### 4.7.2. Spore Germination Inhibition Assay

*FOC* and gradient concentrations of TCX1 were co-cultured at 120 rpm and 25 °C. At 0, 4, 8, 12, 24, and 48 h post-inoculation, 10 μL aliquots were aspirated and transferred to a glass slide to prepare wet mounts. The spore germination rate was immediately assessed under a light microscope (Nikon, Tokyo, Japan). A spore was considered germinated when the germ tube length exceeded half of its diameter. For each replicate, at least 120 spores were counted across random fields of view. The germination rate was calculated as follows: Germination Rate (%) = (Number of germinated spores/Total number of spores counted) × 100. Each treatment had three biological replicates.

### 4.8. FOC Mycelia Membrane Stability Analysis

#### 4.8.1. Assessment of Mycelial Membrane Permeability

*FOC* was cultured in 100 mL of PDB medium at 25 °C with shaking at 120 rpm for 3 d. The mycelia were harvested by centrifugation (8000× *g*, 10 min, 4 °C), washed twice with sterile distilled water, and resuspended in 90 mL of sterile distilled water. This suspension was divided into two groups: (1) the treatment group, which received 10 mL of *Bacillus subtilis* TCX1 suspension (OD_600_ = 1.0); (2) the control group, which received 10 mL of sterile water. The electrical conductivity of the suspension was measured using a conductivity meter (Model DDS-307, INESA Scientific Instrument Co., Shanghai, China) at 4, 8, 12, 24, and 48 h after treatment [52]. Each treatment consisted of three independent biological replicates.

#### 4.8.2. Measurement of MDA Content

The mycelial samples for MDA content determination were the same as those collected for the cell membrane permeability assay. At each time point (4, 8, 12, and 24 h), mycelia were harvested by centrifugation (8000× *g*, 10 min, 4 °C). The malondialdehyde (MDA) content was determined using the thiobarbituric acid (TBA) reaction method as described by Li et al. [53]. Briefly, approximately 0.2 g of ground mycelial tissue (fresh weight) was homogenized in 5 mL of 10% (*w*/*v*) trichloroacetic acid (TCA). The homogenate was centrifuged (12,000× *g*, 15 min, 4 °C). A 2 mL aliquot of the supernatant was mixed with 2 mL of 0.6% (*w*/*v*) TBA solution. The mixture was heated in a boiling water bath for 15 min, quickly cooled on ice, and then centrifuged (10,000× *g*, 10 min) to remove any precipitate. The absorbance of the supernatant was measured at 532 nm, 600 nm, and 450 nm. The MDA concentration was calculated according to the formula provided by Li et al. [53], MDA content (μmol/g FW) = 6.45 × (A_532_ − A_600_) − 0.56 × A_450_, and is expressed as nanomoles per gram of fresh weight. Each treatment consisted of three independent biological replicates.

#### 4.8.3. Determination of Cellular Protein Content

Protein content was measured using the same mycelial samples and sampling time points as described for the cell membrane permeability assay (Section 4.8.1). At each time point, *FOC* mycelia were harvested by centrifugation (12,000× *g*, 20 min, 4 °C). The pellet was resuspended in 5 mL of phosphate buffer (50 mM, pH 7.0) and disrupted by sonication on ice (300 W output, 10 s burst followed by 15 s interval for cooling, repeated for a total of 5 min). The homogenate was centrifuged (12,000× *g*, 20 min, 4 °C) to obtain the clear supernatant for analysis. The protein concentration in the supernatant was determined using the Bradford method with Coomassie Brilliant Blue G-250. Briefly, 100 μL of the supernatant was mixed with 5 mL of Coomassie blue reagent, vortexed thoroughly, and incubated at room temperature for 10 min. The absorbance was then measured at 595 nm. The protein concentration was calculated based on a standard curve prepared with bovine serum albumin (BSA) standards. Each treatment consisted of three independent biological replicates.

### 4.9. Determination of Antioxidant Enzyme Activities

#### 4.9.1. Antioxidant Enzyme Activities in FOC Co-Cultured with TCX1

The mycelia of *FOC* were cultured in 80 mL of PDB medium at 25 °C with shaking at 120 rpm for 3 d. The culture was then supplemented with 10 mL of *Bacillus subtilis* TCX1 suspension (OD_600_ = 1.0) for the treatment group or an equal volume of sterile water for the control group. At 4, 8, 12, 24, and 48 h post-inoculation, mycelia were harvested. A 1.5 g aliquot (fresh weight) was homogenized in 5 mL of ice-cold extraction buffer (0.1 M PBS, pH 7.8, containing 0.0372 g/L EDTA and 10 g/L PVP for SOD, POD, CAT; 0.1 M borate buffer, pH 8.8, containing 4% PVP, 58 mg/L EDTA, and 35 μL/L β-mercaptoethanol for PAL). The homogenate was centrifuged at 10,000× *g* for 20 min at 4 °C, and the supernatant was collected as the crude enzyme extract.

Enzyme activities were determined spectrophotometrically:

SOD activity was determined using the nitroblue tetrazolium (NBT) chloride blue method [54]. One unit (U) of SOD activity was defined as the amount of enzyme that caused 50% inhibition of the NBT reduction rate. The activity was calculated as follows, SOD activity (U/g FW·min) = (D_0_ − D_e_) × V_t_/(0.5 ×W × V_s_), where D_0_ is the absorbance of the control, D_e_ is the absorbance of the sample, V_t_ is the total volume of enzyme extract (mL), W is the fresh weight of the sample (g), and V_s_ is the volume of enzyme extract used in the assay (mL).

POD activity was measured by the guaiacol method at 470 nm. One unit was defined as an increase of 0.01 in absorbance per minute. The activity was calculated as follows, POD activity (U/g FW·min) = (ΔA_470_ × V_t_)/(0.01 × t × W × V_s_), where ΔA_470_ is the change in absorbance per minute, V_t_ is the total volume of enzyme extract (mL), t is the reaction time (min), W is the fresh weight (g), and V_s_ is the volume of enzyme extract used (mL).

CAT activity was determined by monitoring the decomposition of H_2_O_2_ at 240 nm. One unit was defined as the decomposition of 1 μmol H_2_O_2_ per minute. The activity was calculated as follows, CAT activity (U/g FW·min) = (ΔA_240_ × V_t_)/(t × W × V_s_), where ΔA_240_ is the change in absorbance per minute, V_t_ is the total volume of enzyme extract (mL), W is the fresh weight (g), V_s_ is the volume of enzyme extract used (mL), and t is the reaction time (min).

PAL activity was assayed by measuring the formation of trans-cinnamic acid at 290 nm. One unit was defined as the production of 1 μmol trans-cinnamic acid per minute. The activity was calculated as follows, PAL activity (U/g FW·min) = (ΔA_290_ × V_t_ × V_r_)/(ε × d × W × V_s_ × t), where ΔA_290_ is the change in absorbance, V_t_ is the total volume of enzyme extract (mL), V_r_ is the total volume of the reaction mixture (mL), ε is the extinction coefficient of cinnamic acid (9630 M^−1^ cm^−1^), d is the path length (1 cm), W is the fresh weight (g), V_s_ is the volume of enzyme extract used in the assay (mL), and t is the reaction time (min).

All enzyme activities are expressed on a fresh weight basis (U/g FW·min). Each treatment consisted of three independent biological replicates.

#### 4.9.2. Analysis of Antioxidant Enzyme Activities in Cucumber Seedlings

Fresh tissue samples (0.5 g) were homogenized in 5 mL of ice-cold extraction buffer [50 mM phosphate buffer, pH 7.8, containing 1 mM EDTA and 1% (*w*/*v*) polyvinylpolypyrrolidone] and centrifuged at 10,000× *g* for 30 min at 4 °C. The supernatant was collected as the enzyme extract. SOD, POD, and PAL activities were determined using the methods described for *FOC* in Section 4.9.1. PPO activity was assayed by monitoring catechol oxidation at 420 nm. The reaction mixture contained 2.9 mL of 0.1 M phosphate buffer (pH 7.0), 1.0 mL of 0.1 M catechol, and 0.1 mL of enzyme extract. Activity was calculated as PPO activity (U/g FW·min) = (ΔA_420_ × V_t_)/(0.01 × W × V_s_), where V_t_ = total extract volume (mL), W = sample fresh weight (g), and V_s_ = volume of the enzyme extract used in the assay (mL). All activities are expressed on a fresh weight basis. Measurements were performed with three technical replicates per sample. 

### 4.10. Quantitative Real-Time PCR

Total RNA was extracted from cucumber roots, stems, and leaves after 30 d of pot cultivation using the Promega RNA Extraction Kit (Promega Corporation, Madison, WI, USA). According to the instructions of the TaKaRa RT-PCR Kit (TaKaRa Bio Inc., Shiga, Japan) and TaKaRa Taq Kit (R001B, TaKaRa Bio Inc., Shiga, Japan), the total RNA of the samples was reverse transcribed to synthesize cDNA, which was then stored at −20 °C. *Actin* was used as an internal reference gene. Amplifications were performed using a Mastercycler ep realplex2 instrument (Eppendorf, Hamburg, Germany). To calculate the copy numbers of the tested genes, quantitative PCR was used to generate a standard curve for each gene, employing a 10-fold serial dilution of a PCR product as the template. Each measurement was performed in triplicate, and the experiments were repeated three times to ensure consistent results. All primers used in this study are listed in Table S2.

### 4.11. Data Processing and Analysis

Statistical analysis was performed using SPSS 25.0 software (IBM, Armonk, NY, USA). The significance of differences among treatment groups was assessed by one-way analysis of variance (ANOVA). Upon obtaining a significant F-value (*p* < 0.05), the Least Significant Difference (LSD) post hoc test was conducted to compare means between individual groups. All experiments were conducted with three independent biological replicates, and data are presented as means ± standard deviation (SD). Figures were generated using Origin 2021 software (OriginLab, Northampton, MA, USA).

## 5. Conclusions

Inoculation of plants with the endophytic *Bacillus subtilis* strain TCX1 exhibited beneficial effects on plant growth, which could be attributed to its abilities to produce IAA, synthesize siderophores, exhibit ACC deaminase activity, and solubilize mineral phosphates. Additionally, TCX1 enhanced the biocontrol of cucumber *Fusarium* wilt, caused by *FOC*, via multiple mechanisms. These include the production and secretion of cell wall-degrading enzymes such as protease, cellulase, and β-glucanase, and the strain is highly likely to produce and secrete lipopeptides—both of which directly inhibit *FOC*. Furthermore, TCX1 indirectly promoted plant defense by eliciting the expression of antioxidant enzymes and triggering both ISR and SAR in cucumber. This study suggests that the *Bacillus subtilis* strain TCX1 holds significant potential as both a bioagent and a biofertilizer.

## Figures and Tables

**Table 1:** Identification of the endophytic bacteria by 16S rRNA similarity search.

Isolates	Sites	Closest NCBI Match/Species with GenBank Accession Number	Percentage of Identity (%)
TCX1	Root	*Bacillus subtilis* strain Md1-37 (MF581443.1)	99.65
TCX2	Leave	*Pseudomonas fluorescens* strain YG-1 (MN585724.1)	99.86
TCX3	Stem	*Xanthomonas campestris* strain JCT-42 (MF285891.1)	99.79
TCX4	Root	*Lysinibacillus fusiformis* strain BH45 (KY910256.1)	99.72
TCX5	Root	*Pseudomonas aeruginosa* strain M4 (MT180543.1)	100
TCX6	Stem	*Bacillus cereus* strain SRG14 (MK743993.1)	99.93
TCX7	Root	*Enterobacter hormaechei* strain SRG9 (MK743988.1)	99.58
TCX8	Root	*Aeromonas taiwanensis* strain Ichip_2-1 (MW48741 6.1)	99.93
TCX9	Stem	*Curtobacterium flaccumfaciens* pv. *flaccumfaciens* strain x-2 (HQ713508.1)	99.86
TCX10	Root	*Delftia* sp. BN-HKY2 (HQ731449.1)	99.79
TCX11	Root	*Bacillus* sp. strain 6063(MT393628.1)	99.72
TCX12	Stem	*Bacillus safensis* strain JCT-42 (MH820175.1)	99.66
TCX13	Root	*Bacillus safensis* strain KLV20 (MT634636.1)	100
TCX14	Root	*Bacillus pumilus* strain ACCC04398 (MZ067892.1)	99.79

Endophytic bacterial isolates (TCX1-TCX14) obtained from root, stem, and leaf tissues of *Ambrosia artemisiifolia* were identified based on 16S rRNA gene sequence homology using the NCBI BLAST algorithm. The closest matching species, corresponding GenBank accession numbers, and percentage sequence identity are indicated. Strains showing ≥99.5% 16S rRNA gene sequence identity are considered to be identified at the species level.

**Table 2:** Inhibition rate of 5 endophytic bacteria on pathogens.

Endophytic Bacterium	Inhibition Rate (%)				
	TCX1	TCX6	TCX7	TCX12	TCX14
*Acremonium strictum*	–	27	–	–	28.1
*Fusarium graminearum*	66.9	28.3	–	29.7	35.0
*Cercospora zeae-maydis*	57.6	26.0	–	31.7	27.2
*Phytophthora capsici Leonian*	53.7	41.4	37.3	–	–
*Sclerotinia sclerotiorum*	77.0	70.0	36.1	62.5	–
*Bipolaris zeicola*	54.8	63.0	23.2	27.9	31.3
*Fusarium oxysporum* f. sp. *cucumerinum*	86.0	29.6	21.0	45.9	32.8
*Trichothecium roseum*	52.1	33.3	–	74.9	67.8
*Pythium aphanidermatum*	45.0	34.0	30.3	–	36.7
*Fusarium oxysporum* f. sp. *melonis*	71.7	36.0	28.2	53.4	–
*Fusarium culmorum*	49.5	25.6	27.0	24.3	26.0
*Botrytis cinerea Pers*	54.0	–	–	38.5	34.6

Inhibition rates (%) of five endophytic bacterial strains (TCX1, TCX6, TCX7, TCX12, and TCX14) against various plant pathogenic fungi. A dash (“–”) denotes no inhibitory effect.

## Data Availability

All data generated or analyzed in the course of this research are contained within the published article [and its Supplementary Information Files]. For further inquiries, please contact the corresponding author.

## Supplementary Materials

The following supporting information can be downloaded at https://www.mdpi.com/article/10.3390/plants14193068/s1, Table S1: Inhibitory effect of 14 strains of on 12 pathogens; Table S2: Primer sequences used in this research.

## Abbreviations

The following abbreviations are used in this manuscript:

*FOC*	*Fusarium oxysporum* f. sp. *cucumerinum*
SOD	Superoxide dismutase
CAT	Catalase
POD	Peroxidase
PAL	Phenylalnine ammonialyase
PPO	Polyphenol oxidase
CFU	Colony-forming units
OD	Optical density
NCBI	National center for biotechnology information
BLAST	Basic local alignment search tool
MEGA	Molecular evolutionary genetics analysis
PDA	Potato dextrose agar medium
PBS	Phosphate-buffered saline
Rif	Rifampicin
IAA	indole-3-acetic acid
ACC	1-amino-cyclopropane-1-carboxylicacid
MDA	Malondialdehyde
SAR	systemic acquired resistance
ISR	Induced systemic resistance
PCR	Polymerase chain reaction
ROS	reactive oxygen species
RSM	response surface methodology
